# Editorial: Geographic information systems in injury research: bridging spatial science and public health

**DOI:** 10.3389/fpubh.2026.1963939

**Published:** 2026-09-07

**Authors:** Masood Ali Shaikh

**Affiliations:** Department of Medicine, College of Medicine, Korea University, Seoul, Republic of Korea

**Keywords:** clustering detection, disease mapping, geographic information systems, injuries, intimate partner violence, spatial epidemiology

## Introduction

Injury and violence are major public health problems, affecting millions of lives each year. According to the World Health Organization (WHO), violence-related and unintentional injuries account for about 8% of all deaths (4.4 million) and 10% of all years lived with disability globally (1). Thus, imposing a substantial burden on healthcare services and national economies. Violence and injuries are spatially unevenly distributed between and within countries. This Research Topic shows that geographically distinct patterns across outcomes from interpersonal violence and self-harm to musculoskeletal trauma, can inform location-specific prevention and control interventions.

The six articles in this Research Topic show two distinct but complementary approaches of finer-scale identification of place-specific patterns and the broader comparison of geographic burden. The China, Cambodia, and Puerto Rico articles are place-based analyses of intimate partner violence (IPV) or domestic violence. These studies examined spatial clustering, hotspots, and regional variation. The Puerto Rico study examined spatiotemporal non-stationarity as well, showing marked spatial heterogeneity but minimal temporal variation over the 3-year period. The other three articles studied the global burden of self-harm, forearm fracture, and hip dislocation leveraging the Global Burden of Disease (GBD) data, in terms of geographic stratification, temporal trends, and projections for self-harm and hip-dislocation.

Both approaches show patterns in the metrics studied that could be obscured by aggregate estimates. They also highlight high-burden places and demographic groups, even though their spatial resolution, data structures, and statistical techniques differ.

## Contributions

### Theme 1—Mapping and modeling interpersonal violence

#### China: national survey with provincial spatial patterns

This study analyzed data from a large multi-stage survey of 30,505 residents across 148 Chinese cities, aggregated at the provincial level for spatial analyses, and used GIS analyses that entailed global and local spatial autocorrelation and hotspot analyses to study overall, psychological, physical, and sexual IPV (Zhang et al.). Results showed provincial heterogeneity and local clustering, with high burden areas in eastern China. Underscoring the need for spatially nuanced analysis and targeted prevention and response activities.

#### Cambodia: nationally representative cluster-level IPV mapping

This first national spatial analysis of IPV in Cambodia used the 2021–22 Demographic and Health Survey data and combined spatial autocorrelation, Getis-Ord Gi^*^ hotspot analysis, and ordinary kriging to describe geographic variation (Shaikh). IPV was spatially clustered rather than randomly distributed, with concentrations of higher burden in parts of northern Cambodia and lower burden areas elsewhere. The study showed that cluster level mapping can guide prioritization of pertinent services like shelters, counseling, and legal support, among other services, in areas of high need. There is a need to distinguish the observed cluster estimates and statistically identified hotspots from the kriged surface, which is an interpolated approximation.

#### Puerto Rico: spatially and temporally varying domestic-violence associations

The study used police-reported domestic violence data for 13 police regions from 2020 to 2022 and applied geographically and temporally weighted regression (GTWR) to assess how associations with selected sociodemographic factors varied across place and time (Muniu et al.). The study reported spatial clustering in 2020–2021, local hotspots, and geographically varying relationships that a single global regression model could have masked. The temporal variation was minimal over the 3-year period. The locally varying associations can support place-sensitive strategies during situations like emergencies or periods when service access changes. However, the police-reported cases may not necessarily measure the true burden of domestic violence incidence, and the regression findings were only associational and not causal.

### Theme 2—Geographic inequalities in global injury burden and future trends

#### Self-harm: long-term global trends and projections

Using GBD 2021 data, self-harm mortality and disability-adjusted life-year (DALY) burden were examined across 204 countries and territories by age and sex from 1990 to 2021, with projections to 2050 (Xie et al.). The study reported overall declines in age-standardized burden, but with important regional differences. Burden was higher in males, DALY rates peaked in young adults, whereas mortality rates were highest in middle-aged and older populations. Supporting geographically and demographically targeted mental-health and prevention responses.

#### Forearm fractures: counts, standardized rates, causes, and regional disparities

This study characterized global incidence, prevalence, and years lived with disability due to forearm fractures from 1990 to 2021, including variations by region, sex, age, and cause of injury (Fan et al.). While absolute numbers increased, age-standardized rates declined, with notable regional and sex disparities. Highlighting the growing absolute service demand, partly owing to population growth and aging. Falls were identified as a major cause of these fractures, so fall prevention, trauma services, and osteoporosis-related services require region-specific resource planning and response.

#### Hip dislocation: demographic and geographic burden with projections

Using GBD 2021 data, burden estimates, Socio-Demographic Index-based ecological comparisons, and Bayesian age-period-cohort projections were presented to describe hip-dislocation burden through 2021, with projections to 2030 (Zheng et al.). Increasing case counts but declining age-standardized rates, higher burden in males, and important peaks in younger and older populations were reported. The geographic and demographic patterns reflect the need for prevention of falls and road trauma, timely access to orthopedic care, rehabilitation capacity, and planning for different age and risk settings.

## Cross-cutting lessons, limitations, and future priorities

Collectively across the six studies, the geographically stratified metrics identify where more granular GIS analyses, surveillance strengthening, access studies, and importantly, locally tailored prevention and control efforts are needed. Because geographic context can change interpretation, the research question needs to determine spatial scale. Incorporating time can make place-based analyses more informative, and demographic and geographic inequities often intersect across places.

Limitations and caveats include under-reporting of violence, survey GPS-coordinate displacement and geographic aggregation, sparse local observations, inconsistent data quality between settings, ecological inference, and uncertainty in modeled estimates and projections. To improve equity, spatial analysis should move from descriptive mapping toward evaluation of geographically targeted interventions to improve prevention, access, response time, service coverage, and health outcomes.

## Conclusion

National/provincial description in China, cluster-level hotspot and interpolation analysis in Cambodia, and localized spatiotemporal regression in Puerto Rico answer different public-health questions and operate at different spatial scales. The GBD articles distinguish changes in absolute burden from changes in standardized rates and show how global improvement can coexist with high or rising needs in particular regions, age groups, and sexes. Location and scale reveal nuanced burden and suggest location-specific interventions across outcomes from interpersonal violence and self-harm to musculoskeletal trauma. The Research Topic demonstrates the value of spatial perspectives across diverse injury and violence outcomes, methods, and geographic scales. However, spatial evidence has greater utility when it informs geographically tailored prevention and control responses and priorities.

## References

[1] World Health Organization. Injuries and Violence [Internet]. Geneva: World Health Organization (2024). Available online at: https://www.who.int/news-room/fact-sheets/detail/injuries-and-violence (Accessed August 04, 2026).

